# Minimally invasive versus transcatheter closure of secundum atrial septal defects: a systematic review and meta-analysis

**DOI:** 10.1177/02676591211021935

**Published:** 2021-06-10

**Authors:** Esther Goh, Haya Mohammed, Mohammad Yusuf Salmasi, Samantha Ho, Umberto Benedetto, Massimo Caputo, Gianni Angelini, Hunaid A Vohra

**Affiliations:** 1 Department of Cardiovascular Sciences Surgery, Bristol Hearth Institute, Bristol, UK; 2 Surgery and Cancer, Imperial College London, London, UK

**Keywords:** minimally invasive, transcatheter, atrial septal defect

## Abstract

**Background:**

Limited data exists demonstrating the efficacy of minimally invasive surgery (MIS) compared to transcatheter (TC) closure of atrial septal defects (ASD). This systematic review and meta-analysis aims to compare post-operative outcomes of MIS versus TC repair in ASD closure.

**Methods:**

PubMed, Medline and EMBASE were searched from inception until June 2018 for randomised and observational studies comparing post-operative outcomes for MIS and TC repair. The studies were reviewed for bias using the ROBINS-I Score and pooled in a meta-analysis using STATA (version 15).

**Results:**

Six observational studies, involving 1524 patients assessing three primary and five secondary outcomes were included. Evidence suggests TC repair yielded shorter hospital stay (MD = 3.32, 95% CI 1.04–5.60) and lower rates of transient atrial fibrillation (AF) (RR = 0.48, 95% CI 0.20–1.15). TC repair patients also had fewer pericardial effusions (RR = 0.27, 95% CI 0.05–1.54, I^2^ = 0.0%) and pneumothoraxes (RR = 0.18, 95% CI 0.04–0.80, I^2^ = 0.0%). However, TC repair results in more minor residual shunts (RR = 6.04, 95% CI 1.69–21.63 in favour of MIS, I^2^ = 39.0%). No differences were found for incidences of strokes (RR = 1.58, 95% CI 0.23–10.91, I^2^ = 19.3%), unexpected bleeding (RR = 0.44, 95% CI 0.19–1.04, I^2^ = 0.0%) and blood transfusion (RR = 0.39, 95% CI 0.09–1.59, I^2^ = 0.0%).

**Conclusions:**

MIS closure for ASD has similar outcomes compared to TC repair. However, the lack of randomised literature related to MIS versus TC repair for ASD closure warrants further evidence in the form of RCTs to further support these findings.

## Introduction

Atrial Septal Defects (ASDs) are one of the most common congenital cardiac defects.^1^ In children, they are often asymptomatic and can be overlooked; however, a long-term shunt can cause pulmonary over-circulation due to left-to-right shunting.^2^ As a result, pulmonary vascular remodelling and increased preload can occur, predisposing the patient to heart failure, atrial arrhythmias, thrombosis and paradoxical embolisms.^3,4^ Nonetheless, if corrected early enough, the heart can restore its normal physiological state, thereby avoiding these complications.^5–7^ Therefore, proper closure of the shunt is essential and can be achieved spontaneously or through intervention. A 2006 study of 200 patients reported spontaneous closure in 56% of patients with defects from 4 to 5 mm, whereas no spontaneous closure occurred in large defects (>10 mm).^8^ As a result, a wait-and-see approach is adopted for small defects, whilst interventional or surgical closure is preferred for larger defects.

There are three common ways to treat ASDs: median full sternotomy, minimally invasive surgery (MIS) and transcatheter (TC). A median full sternotomy is the conventional approach due to its simplicity, low mortality and morbidity rates.^9,10^ The procedure is reliable; however, it is associated with cosmetic concerns and several potential complications due to the use of cardiopulmonary bypass (CPB) such as atrial fibrillation, pericardial effusions and pneumothoraxes. Minimally invasive cardiac surgery was introduced in 1953 and has similar outcomes as median sternotomy.^11^ There is a steeper learning curve with key hole surgery, but it allows for less postoperative pain, shorter hospital stays and an earlier return to physical activity.^12^ Despite the procedure being aesthetically more pleasing, it shares the consequences that arise from the use of CPB.

The TC closure was introduced in 1974 with the aim to further reduce the length of hospital stay (LOHS) and encourage early mobilisation, while removing the need for CPB.^13^ However, not all patients are suited for this method. Patients must not have multiple ASDs, have an ASD <38 mm, and have enough rims (>5 mm) to qualify for a percutaneous closure.^14–16^ The most commonly used device is the Amplatzer Septal Occluder (St. Jude Medical, St. Paul, MN, USA).^17^ The Amplatzer is designed to promote occlusion and tissue-in-growth which suggests that the device becomes more effective the longer it is in place.^18^ In this meta-analysis, we aim to compare the post-operative outcomes of MIS with TC for ASD closure.

## Method

### Search methodology

Online databases PubMed, MEDLINE Ovid and EMBASE were extensively searched for studies published in English from their dates of inception to June 2018 using combined terms of: ‘transcatheter’, ‘minimally invasive’ and ‘atrial septal defects’ in humans in accordance with recommendations by PRISMA guidelines (Appendix 1). Resultant studies were distributed amongst authors for title and abstract screening according to a predefined inclusion criteria. Outcomes of length of hospital stay (LOHS), evidence of residual shunts, atrial fibrillation (AF), stroke, pericardial effusion, pneumothorax, bleeding and requirement of blood products were included. Reference lists of the selected articles were further reviewed to identify any other relevant studies.

### Selection criteria

**Figure 1.**
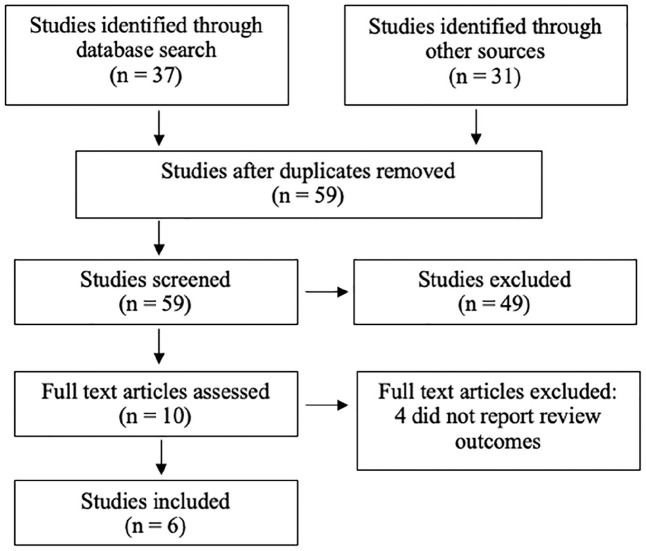
PRISMA flow chart outlining the search and study selection.

Retrospective and observational studies were used to compare the complications and outcomes for ASD repairs via MIS and TC repair since no randomised controlled trials exist. Studies which evaluated conventional midline sternotomy for closure of ASDs and did not compare the two modes of interest (TC and MIS), or were published as case reports, editorials or commentaries were excluded (Figure 1). The exact approach used in MIS surgery was not restricted and included thoracoscopic, thoracotomy or mixed techniques. Methods of ASD closure in both TC and MIS groups included device implantation, patch or suture closure, which were not controlled since our interest involved comparing the routes of ASD closure rather than which technique was used to close the ASD.

Two reviewers (H.M, E.G) independently conducted the literature search and assessed titles and abstracts for selection. To be included, the study must have directly compared MIS to TC repairs and have reported the chosen outcomes mentioned above. Any discrepancies were resolved through discussion with a third party to achieve a consensus. A data extraction form was used to retrieve study characteristics and outcome data. The majority of variables were reported as means ± standard deviation (*SD*) or number (*n*) and percentage (%). However, when data was reported as medians with interquartile ranges, the Cochrane guidelines were used to express these values into means with SD.

### Assessment of risk of bias in studies

Risk of bias of the pooled non-randomised studies was assessed using the ROBINS-I tool^19^ (Appendix 2). The studies were assessed twice by H.M, E.G and S.H. Any discrepancies were discussed, re-assessed and settled where necessary with a senior author (H.V).

### Measurement of treatment effect

Meta-analysis of the outcomes data was conducted using STATA (version 15) to create the forest plots. Continuous data was analysed as a means difference with 95% confidence intervals. Pooled data was presented as a risk ratio (RR). The data was then weighted for random effects. Therefore, the analysis not only considers the statistical heterogeneity of the individual study, which is mainly governed by the number of participants, but also the variance between the studies.

## Results

### Results of the search

A total of 37 studies were retrieved through the electronic search of databases. An additional 31 studies were found through other sources including informal searches and references lists. After reviewing titles and abstracts, 62 studies were excluded, and 6 full texts were assessed (Figure 1). The six studies observed a total of 1534 patients who had undergone either a TC repair (895 patients) or MIS (639 patients) procedure to correct an ASD (Table 1).

**Table 1.** Pre-operative characteristics of included studies.

Author (ref no.)	Country	Study period	Study design	Total *n*	*n* (TC)	*n* (MIS)	Mean follow-up (months)	Group	Mean age (years)	Female, *n* (%)	NYHA ⩾ 3 (%)	HTN (%)	DM (%)
Bakar et al.^20^	Canada	2009–2017	RSP	61	28	33	0.25–89	TC	57	18 (64)	1 (4)	10 (36)	3 (11)
								MIS	37	24 (73)	2 (6)	6 (18)	2 (6)
Formigari et al.^21^	Italy	1996–1998	RSP	123	52	71	18–41	TC	7				
								MIS	5				
Guo et al.^22^	China	2003–2009	RSP	92	42	50	1–12	TC	34	26 (62)			
								MIS	38	32 (64)			
Kodaira et al.^23^	Japan	2000–2003	ORSP	354	134	220	6–48	TC	52 ± 20	82 (61)		23 (17)	7 (5)
								MIS	41 ± 16	165 (75)		18 (8)	5 (2)
Mishra et al.^24^	India	1997–2006	RSP	640	470	170	0	TC	26	263 (56)			
								MIS	34	98 (58)			
Schneeberger et al.^25^	Germany	2002–2014	ORSP	264	169	95	1–12	TC	50 ± 16	128 (76)	0		16 (9)
								MIS	38 ± 13	64 (67)	0		2 (2)

DM: diabetes; HTN: hypertension; MIS: minimally invasive surgery; NYHA: New York Heart Association; ORSP: observational retrospective study; RSP: retrospective; TC: transcatheter.

The characteristics of the six included studies are summarised in Table 1.^20–25^ It is worth noting that the studies were all single centred and had a variable mean patient age. This difference in mean ages was accounted for during data analysis. Additionally, the length of follow-up was inconsistent with the longest being 89 months, therefore limiting the evaluation of persistent outcomes of surgery. Peri-operative and post-operative characteristics are demonstrated in Tables 2 and 3.^20–25^

**Table 2.** Procedural and intra-operative outcomes of the included studies in our review.Peri-operative characteristics of included studies.

Author (ref no.)	Group	ASD size (mm)	ASD type			Conversion to other procedure	Incidence of dislodgment (%)
			Secundum	Patent Foramen Ovale	Other (sinus venosus, partial AVSD, unroofed CS)		
Bakar et al.^20^	TC	16.5	27 (96%)	1 (4%)	0		
	MIS	23.5	26 (79%)	3 (9%)	7 (12%)		
Formigari et al.^21^	TC	12 ± 4	52 (100%)	0	0	2 (4%), converted to sternotomy	2 (4)
	MIS		71 (100%)	0	0		
Guo et al.^22^	TC	31 ± 3	42 (100%)	0	0	1 (2%), converted to sternotomy	1 (2)
	MIS	30 ± 3	50 (100%)	0	0	1 (2%), converted to sternotomy	1 (2)
Kodaira et al.^23^	TC	18 ± 6	134 (100%)	0	0	2 (1%), converted to MIS	
	MIS	21 ± 8	220 (100%)	0	0	0	
Mishra et al.^24^	TC		0	461 (98%)^a^	0	13 (3%), converted to sternotomy	9 (2)
	MIS		0	169 (99%)^a^	0	1 (0.6%), converted to sternotomy	0
Schneeberger et al.^25^	TC		169 (100%)	0	0	0	1 (0.6)
	MIS		84 (88%)	0	11 (12%)	0	0

ASD: atrial septal defect; AVSD: atrioventricular septal defect; CS: coronary sinus; MIS: minimally invasive surgery; TC: transcatheter.

a Dropped out because in TC devise closure was not suitable and in MIS femoral artery was too small.

**Table 3.** Early post-operative outcomes of the included studies.Post-operative outcomes in included studies.

Author (ref no.)	Group	Length of hospital stay (days)	Length of ICU stay (hours)	Residual shunt (%)	AF (%)	Stroke (%)	Pericardial effusion (%)	Pneumothorax (%)	Bleeding (%)	Blood product use (%)
Bakar et al.^20^	TC	1	0	4 (14)	5 (18)	1 (4)			1 (4)	0
	MIS	5	24	2 (6)	5 (15)	0			0	1 (3)
Formigari et al.^21^	TC	2.1		4 (8)				0		
	MIS	2.8		1 (1)			1 (1)	1 (1)	1 (1)	
Guo et al.^22^	TC	7.5		4 (9)	2 (5)	0	0	0	1 (2)	1 (2)
	MIS	11.9		3 (6)	3 (6)	0	2 (4)	1 (2)	2 (4)	2 (4)
Kodaira et al.^23^	TC	3.6			0	0	0	0		1 (1)
	MS	7.3			1 (1)	3 (1)	3 (1)	8 (4)		5 (2)
Mishra et al.^24^	TC	1.1	6–8	12 (2.6)	4 (1)					
	MIS	6	67.2	1 (1)	1 (1)			1 (1)		
Schneeberger et al.^25^	TC			0	9 (10)	1 (1)^a^				
	MIS	6.1	36	9 (9)^a^	0^a^	0				

AF: atrial fibrillation; ICU: intensive care unit; MIS: minimally invasive surgery; TC: transcatheter.

a Matched group data.

### Results of bias assessment of included studies

**Figure 2.**
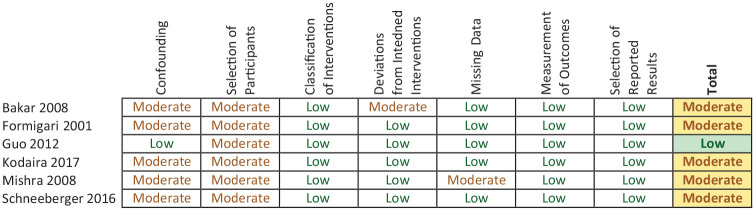
Risk of bias summary table.

The assessment found one study was low bias, and five were moderate bias. In regard to patient selection, all studies were of moderate bias because of the retrospective nature of the studies and the patient requirements for the TC repair. The ‘Risk of Bias Summary’ provides a breakdown of the different domains (Figure 2).

### Results of interventions

#### Hospital and ICU Stay

**Figure 3.**
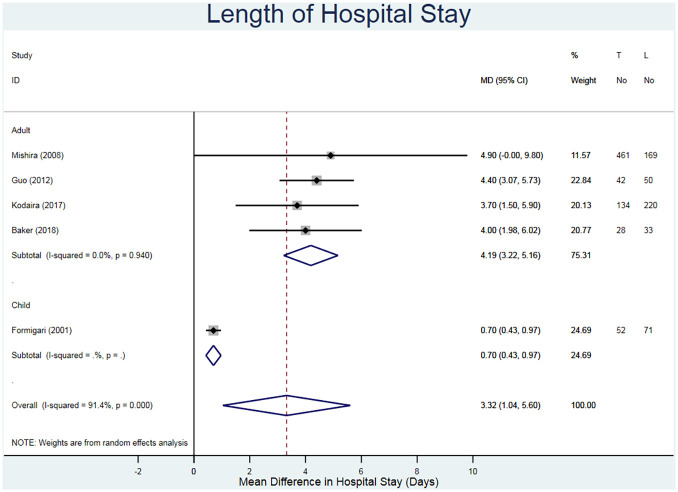
Forest plot for the length of hospital stay. Pooled mean difference and 95% CI is presented by the centre and tips of the diamond. Squares and horizontal lines show effect estimate and 95% CI for individual studies.

The LOHS was reported for 1534 adults and 93 children across the studies (Table 3).^20–25^ Schneeberger et al.^25^ reported only the LOHS for MIS, hence it was excluded. The overall effect estimate suggests that the TC repair has a lower LOHS regardless of the age of patient (MD = 3.32 days, 95% CI 1.04–5.60). When solely considering adults, the difference between the LOHS for each intervention increased (MD = 4.19 days, 95% CI 3.22–5.16) (Figure 3). Heterogeneity was unremarkable (I^2^ = 0.0%) when solely considering the studies with adults. However, it became significant (I^2^ = 91.4%) once the study by Formigari et al.^21^ was added due to the large difference in age between the populations of the studies. Additionally, three studies evaluated the intensive care unit (ICU) stay (Table 3).^20,24,25^ Bakar et al.^20^ recorded the mean ICU stay of MIS patients as 1 day with an IQR of (1–1) in comparison to TC repair patients, of which none were admitted to ICU. This is in line with Mishra et al.^24^ who reported a mean of 2.8 days (1–10 days) in ICU for the MIS patients versus a mean of 6–8 hours for the TC repair patients, and Schneeberger et al.^25^ who reported a mean of 1.5 days ±0.8) for the MIS patients.

#### Residual shunts

**Figure 4.**
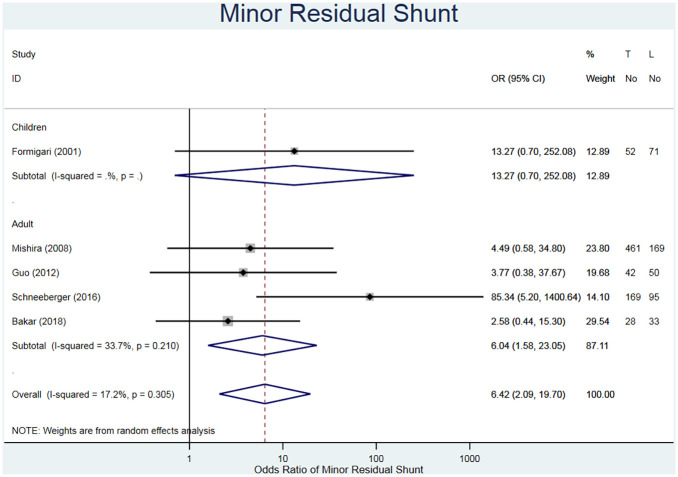
Forest plot for minor residual shunts. Pooled odds ratio and 95% CI is presented by the centre and tips of the diamond. Squares and horizontal lines show effect estimate and 95% CI for individual studies.

Major residual shunts defined the success of the procedures. The studies classified shunts as major or minor depending on whether there was a functional consequence of the shunt. Patients were assessed immediately after and during follow-ups using colour Doppler flow imaging.^22^ Aside from Kodaira et al.^23^ who did not evaluate residual shunts, no major residual shunts were reported in any of the studies. Therefore, regardless of the number of minor residual shunts, by definition both interventions are considered reasonable options for ASD closure. Minor residual shunts were reported in 1180 adults across five studies (Table 3).^20–22,24,25^ The studies are separated based on children or adults, with an overall risk ratio of a resulting minor residual shunt displayed (RR = 6.04, 95% CI 1.69–21.63 in favour of MIS approach, I^2^ = 39.0%) (Figure 4). The large individual 95% CIs are due to low numbers in both the study populations and the number of patients. It is worth mentioning that the CIs for the studies of Formigari et al.^21^ and Schneeberger et al.^25^ are attributed to the fact that there were no cases of minor residual shunts in the MIS group.

#### Atrial fibrillation

**Figure 5.**
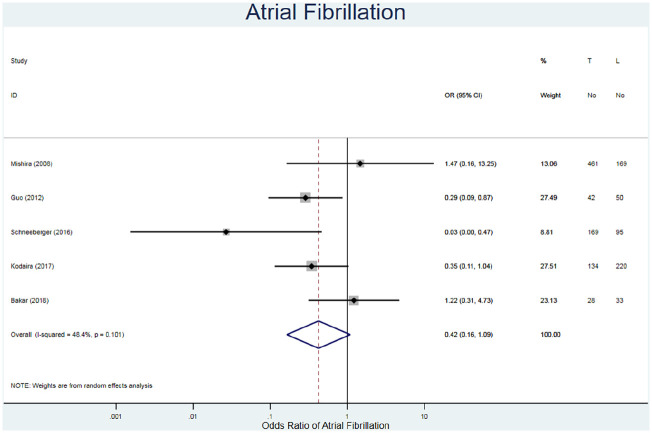
Forest plot for atrial fibrillation. Pooled odds ratio and 95% CI is presented by the centre and tips of the diamond. Squares and horizontal lines show effect estimate and 95% CI for individual studies.

The presence of transient atrial fibrillation (AF) was reported for 1411 adults across five studies (Table 3).^20,22–25^ Overall the risk of transient AF are higher in the MIS group (RR = 0.48, 95% CI 0.20–1.15) (Figure 5), however it worth noting that it is not significant against the null hypothesis. Two of the studies reported a higher percentage in patients after the MIS procedure.^22,23^ On the other hand, Bakar et al.,^20^ Mishra et al.^24^ and Schneeberger et al.^25^ recorded higher percentages in the TC repair method. This alludes to the possibility of both methods being susceptible to AF.

#### Stroke

**Figure 6.**
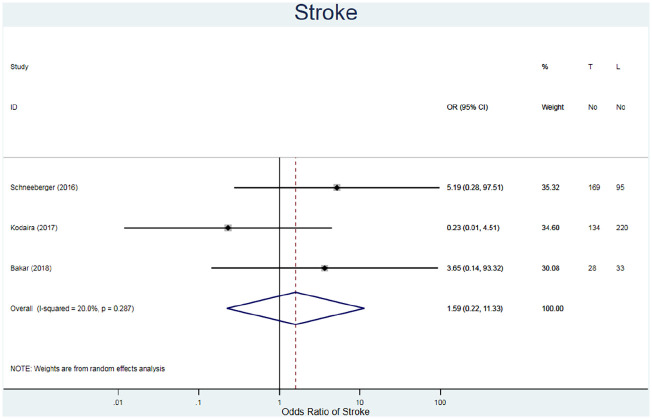
Forest plot for stroke. Pooled odds ratio and 95% CI is presented by the centre and tips of the diamond. Squares and horizontal lines show effect estimate and 95% CI for individual studies.

The incidence of strokes was reported for 679 patients in three studies (Table 3).^20,22,25^ The fixed risk ratio favours the MIS approach regarding having a stroke post operatively (RR = 1.58, 95% CI 0.23–10.91, I^2^ = 19.3%) (Figure 6). The confidence intervals for all the studies are wide due to the small number of events that occurred and the absence of events in the other cohort. Additionally, there is no further information as to the timing of the strokes (immediately after the procedure or up to months after) or any preventative measures, such as anticoagulation regimes, undertaken.

#### Pericardial effusion

**Figure 7.**
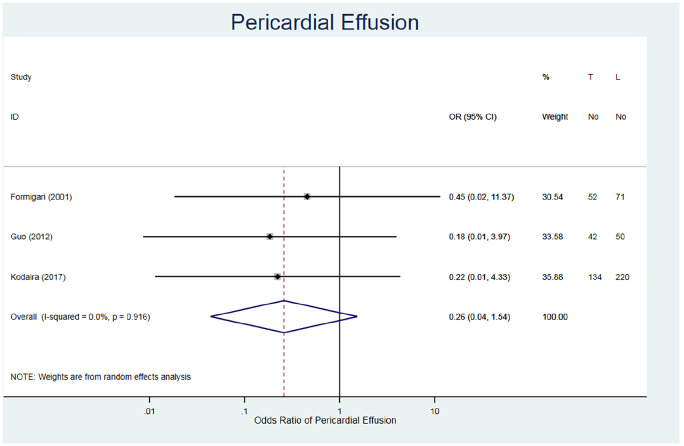
Forest plot for pericardial effusion. Pooled odds ratio and 95% CI is presented by the centre and tips of the diamond. Squares and horizontal lines show effect estimate and 95% CI for individual studies.

The incidence of pericardial effusion was evaluated in three studies for 569 patients (Table 3).^21–23^ The fixed risk ratio demonstrates a lower rate of pericardial effusion post-operatively in TC repair patients (RR = 0.27, 95% CI 0.05–1.54, I^2^ = 0.0%) (Figure 7). Additionally, there are wide 95% CIs across the studies, which all cross the line of no effect. Therefore, there is no statistically significant difference between TC repair and MIS on post-operational pericardial effusion.

#### Pneumothorax

**Figure 8.**
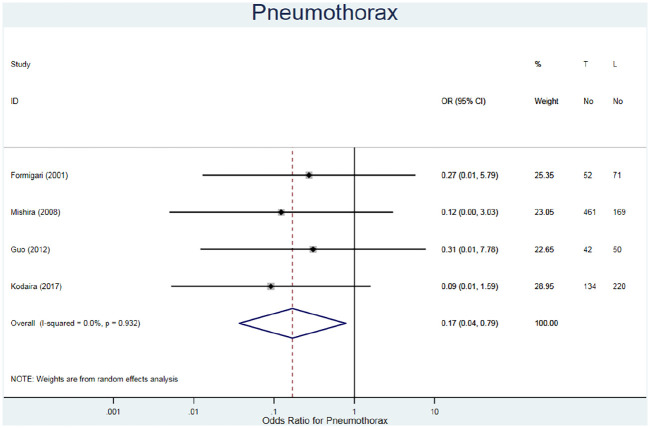
Forest plot for pneumothorax. Pooled odds ratio and 95% CI is presented by the centre and tips of the diamond. Squares and horizontal lines show effect estimate and 95% CI for individual studies.

Pneumothorax was evaluated by four studies for 1209 patients (Table 3).^21–24^ Similar to pericardial effusions, the fixed risk ratio demonstrates a lower rate in TC repair patients (RR = 0.18, 95% CI 0.04–0.80, I^2^ = 0.0%) (Figure 8). It should be considered that none of the studies had any events of pneumothorax in the TC repair cohorts, therefore resulting in large 95% confidence intervals.

#### Bleeding

**Figure 9.**
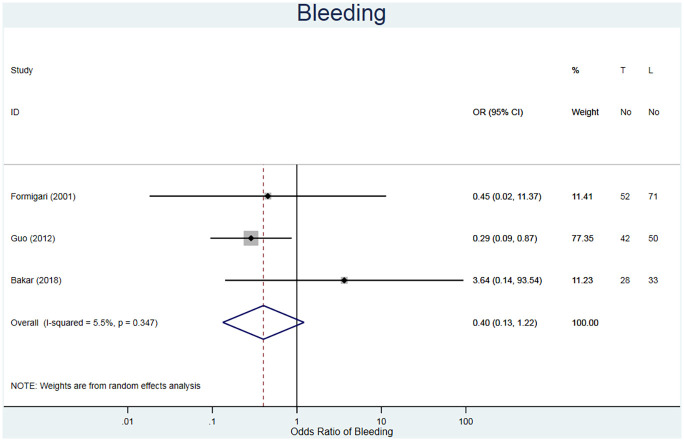
Forest plot for excessive bleeding. Pooled odds ratio and 95% CI is presented by the centre and tips of the diamond. Squares and horizontal lines show effect estimate and 95% CI for individual studies.

The incidence of unexpected bleeding was studied for 276 patients in three studies (Table 3).^20–22^ The fixed odds ratio is in favour of the TC repair method (RR = 0.44, 95% CI 0.19–1.04, I^2^ = 0.0%) (Figure 9). Although reported, none of the studies provided criteria to define bleeding or the extent of bleeding; therefore, the validity of the comparison is uncertain.

#### Requirement of blood products

**Figure 10.**
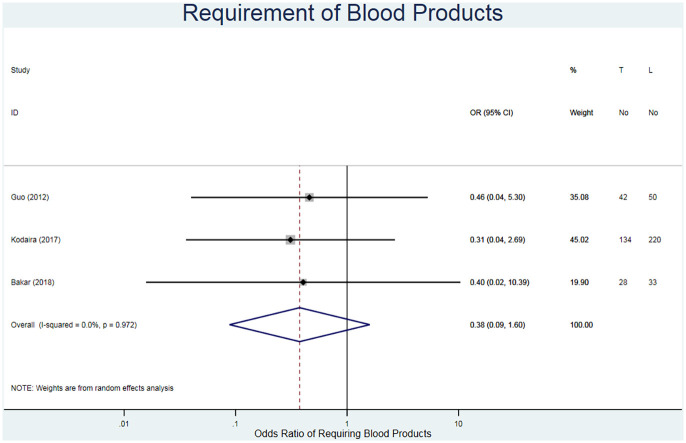
Forest plot for requirement of blood products. Pooled odds ratio and 95% CI is presented by the centre and tips of the diamond. Squares and horizontal lines show effect estimate and 95% CI for individual studies.

The requirement of blood products refers to the unplanned use of blood products throughout the procedure. This was reported for 507 patients across three studies (Table 3).^20,22,23^ The fixed odds ratio is in favour of the TC repair method (RR = 0.39, 95% CI 0.09–1.59, I^2^ = 0.0%) (Figure 10).

## Discussion

This systematic review and meta-analysis comparing MIS with TC repair for ASD closure highlights the patient-specific advantages and disadvantages of each method. It shows that the LOHS is significantly shorter in the TC repair method. The MIS method requires multiple small incisions as well as cardio-pulmonary bypass (CPB) and handling of the myocardium.^11,20^ On the other hand, in the TC repair method the device is inserted into a vein through the groin; there are fewer incisions and no need for CPB.^16^ This may explain quicker patient recovery and avoid the risks associated with long-term bed rest. Neither methods resulted in any major residual shunts, however, the incidence of minimal residual shunts was higher in the TC repair method, the clinical significance of which is uncertain. While MIS repair of ASD is tailored to each shunt by using either direct sutures or a patch under direct or endoscopic vision, the TC repair method uses a generic device that may not immediately conform to the atrial septum completely.^11,20,23^ However, tissue growth around the device in due course^18^ is believed to reduce the long-term residual shunting.^17^ Post-procedural AF was greater in the MIS group, which is in line with previous reports.^26^ It has been found that the external handling of the myocardium results in changes that can induce atrial arrhythmias. This is mainly avoided in the TC repair method. Despite this, the incidence of strokes was similar between the two groups. Pericardial effusions and pneumothoraxes were only recorded in the MIS group. These complications have been found to be associated with right thoracotomies.^23^ This correlation has been linked to the need for CPB, which explains the absence in the TC repair patients.^27^ Finally, both outcomes of excessive bleeding and blood product usage were in favour of the TC repair method. It is also worth noting that there was no occurrence of other complications that required a conversion to a median sternotomy or re-operation.

This systematic review and meta-analysis is based on single centre, retrospective studies, which are prone to selection bias and inconsistent follow-up; therefore, the overall quality of evidence may not be optimal.^28^ As none of the studies had long-term follow-up, we were unable to evaluate complications that would have arisen because of the device such as allergies, cardiac erosion and device embolism.^23^ We have shown, based on the evidence available with the help of a systematic review and meta-analysis that the TC repair method is associated with (1) significantly lower LOHS, (2) lower rates of post-operative AF and (3) fewer incidents of major post-operative complications such as pericardial effusion or pneumothorax. The TC repair method uses a fixed device, hence limiting its availability to patients due to anatomical variation, and results in a higher risk of small residual shunts.

In conclusion, both MIS and TC repairs are suitable for ASD closure, each having their own advantages and disadvantages; however, there is insufficient evidence to state that one is clearly superior to the other. This paper demonstrates the need for further randomised comparison between MIS and TC repair with larger sample sizes and long term follow up.

## Supplemental Material

sj-pdf-1-prf-10.1177_02676591211021935 – Supplemental material for Minimally invasive versus transcatheter closure of secundum atrial septal defects: a systematic review and meta-analysis Click here for additional data file. Supplemental material, sj-pdf-1-prf-10.1177_02676591211021935 for Minimally invasive versus transcatheter closure of secundum atrial septal defects: a systematic review and meta-analysis by Esther Goh, Haya Mohammed, Mohammad Yusuf Salmasi, Samantha Ho, Umberto Benedetto, Massimo Caputo, Gianni Angelini and Hunaid A Vohra in Perfusion
